# Internalin AB-expressing recombinant *Lactobacillus casei* protects Caco-2 cells from *Listeria monocytogenes*-induced damages under simulated intestinal conditions

**DOI:** 10.1371/journal.pone.0220321

**Published:** 2019-07-29

**Authors:** Moloko G. Mathipa, Arun K. Bhunia, Mapitsi S. Thantsha

**Affiliations:** 1 Department of Biochemistry, Genetics and Microbiology, University of Pretoria, Pretoria, South Africa; 2 Molecular Food Microbiology Laboratory, Department of Food Science, Purdue University, West Lafayette, Indiana, United States of America; 3 Department of Comparative Pathobiology, Purdue University, West Lafayette, Indiana, United States of America; 4 Purdue Institute of Inflammation, Immunology, and Infectious Disease, Purdue University, West Lafayette, Indiana, United States of America; Institute of Molecular Genetics and Genetic Engineering, SERBIA

## Abstract

**Background:**

*Listeria monocytogenes* is an intracellular foodborne pathogen that employs a number of strategies to survive challenging gastrointestinal conditions. It proliferates in the gut and subsequently causes listeriosis in high-risk individuals. Therefore, inhibition of its adherence to the intestinal receptors is crucial in controlling its infection. In this study, the effect of our previously developed recombinant *Lactobacillus casei* strain expressing invasion protein, Internalin AB of *L*. *monocytogenes* (Lbc^InlAB^) on epithelial infection processes of the latter under simulated intestinal conditions was investigated.

**Materials and methods:**

The confluent Caco-2 cell monolayer was pre-exposed to different *L*. *casei* strains at a multiplicity of exposure (MOE) of 10 for various periods before infection with *L*. *monocytogenes* at a multiplicity of infection (MOI) of 10 under simulated intestinal conditions. Subsequently, *L*. *monocytogenes* adhesion, invasion, and translocation, cytotoxicity and impact on tight junction integrity of the Caco-2 cells were analyzed.

**Results:**

Under the simulated gastrointestinal condition, Lbc^InlAB^ showed a significant increase (p<0.0001) in adherence to, invasion and translocation through the Caco-2 cells when compared with the wild type strain. Although Lbc^InlAB^ strain exhibited enhanced inhibition of *L*. *monocytogenes*, it was not able to displace *L*. *monocytogenes* cells already attached to the monolayer. Additionally, pre-exposure to Lbc^InlAB^ reduced *L*. *monocytogenes*-mediated cytotoxicity and protected the tight junction barrier function.

**Conclusion:**

The recombinant *L*. *casei* expressing InlAB shows potential for use as a prophylactic intervention strategy for targeted control of *L*. *monocytogenes* during the intestinal phase of infection.

## Introduction

*Listeria monocytogenes* is abundant in nature and has proven to proliferate under various environmental conditions [1] including temperatures between -1.5 to 45°C and broad pH range of 4.0 to 9.6. It is well suited to survive in foods, and transit through the gastrointestinal tract. In immunocompromised individuals and pregnant women, it causes a disease generally referred to as listeriosis, while it results in self-limiting gastroenteritis in healthy individuals. *L*. *monocytogenes* employs different survival strategies in the challenging microenvironments of the gastrointestinal tract [2–4]. It enters the body through gastrointestinal mucosal surfaces to cause infection [5]. During disease progression, *L*. *monocytogenes* employs the *Listeria* adhesion protein (LAP), to aid adhesion and transmigration across the epithelial barrier in the gut [6,7]. It also uses the surface protein Internalin A (InlA) [8,9] and InlB [10,11] to attach to and gain entry into host cells for systemic spread. This invasion has also been shown to be associated with murine M cells both *in vivo* and *in vitro* [12].

Attenuated strains of foodborne pathogens such as *L*. *monocytogenes* have been used as vaccines for their control. However, such strains present the two most important risks. Firstly, the attenuated strain has a potential for reversion to its virulent phenotype post administration. Secondly, they can be virulent in partially immunocompetent (young infants; elderly) or immunocompromised individuals as they retain residual virulence [13]. These risks prompted an interest in a search for alternative strategies for pathogen control. Non-pathogenic transient bacteria in the digestive tract were then suggested as an alternative that can be used to substitute the attenuated pathogens [14,15]. Probiotics are candidates of choice as they have been reported to confer a health benefit on the host (FAO/WHO, 2002) [16] and to competitively inhibit foodborne pathogen infection [17,18]. Their advantages include tolerance to acid and bile salts allowing for survival, transition through the gastrointestinal tract (GIT) and colonization of the mucosal surface [19]. However, several studies have reported that probiotics sometimes fail to produce desirable effects. McCarthy *et al*. [20] reported the same levels of the anti-inflammatory cytokine TGF-β in untreated mice suffering from colitis and those to which probiotic *Lactobacillus salivarius* and *Bifidobacterium infantis* were orally administered. Koo *et al*. [21] reported inefficiency of wild type probiotic strains in preventing the attachment of *L*. *monocytogenes* to intestinal epithelial monolayers *in vitro*.

In an effort to enhance the effectiveness or antipathogenic effects of probiotics, bioengineering has been used as an alternative strategy [19,22], where a virulent gene of the specific pathogen is cloned and expressed to create a recombinant probiotic strain that is subsequently used to competitively exclude that pathogen. The bioengineered probiotics were reported by different researchers to possess enhanced antipathogenic effects when compared to their wild-type counterparts [21,23,24]. Chu *et al*. [23] reported the ability of a recombinant *L*. *acidophilus* strain carrying the K99 fimbriae from enterotoxigenic *Escherichia coli* (ETEC) to reduce attachment of ETEC to the porcine intestinal brush border in a dose-dependent manner. In another study, Wu *et al*. [25] cloned and expressed the heat stable (ST) and heat labile (LT) enterotoxins of ETEC under the nisin-inducible promoter into probiotic *Lactobacillus reuteri*. They reported that the recombinant strain successfully prevented enterotoxicity in a mouse model. Koo *et al*. [21] cloned and expressed the *Listeria* adhesion protein (LAP) from *L*. *monocytogenes* into probiotic *L*. *paracasei* and demonstrated that the recombinant strain was able to exclude *Listeria* colonization and epithelial cell damage *in vitro*. Depending on the virulence genes that are cloned and expressed, these genetically engineered probiotics can be used either as prophylactic or treatment alternatives for the specific pathogens. Due to promising results reported in these aforementioned studies, we also recently cloned and expressed the invasion operon *inlAB* into *Lactobacillus casei* (Lbc^InlAB^) for the inhibition of *L*. *monocytogenes* adhesion, invasion and translocation *in vitro*, using the Caco-2 cells grown and maintained in the cell culture media, Dulbecco's Modified Eagle's Medium (DMEM) supplemented with fetal bovine serum (FBS) [26]. The resultant recombinant probiotic showed enhanced inhibition of *L*. *monocytogenes* compared to the wild type *L*. *casei*. Although the results were positive, they were still not appropriate for making inferences about how the recombinant *L*. *casei* would affect the *L*. *monocytogenes* intestinal infection phase, as the media used did not sufficiently simulate the intestinal conditions.

The Caco-2 cell line, a colon adenocarcinoma cell origin differentiates into the enterocyte-like cell and is a widely used model to assess intestinal permeability [27,28]. Different media such as the buffered salt solution, Hanks’ balanced salt solution (HBSS) buffered with HEPES (10 mM) at pH 7.4, and the DMEM containing amino acids and vitamins, both supplemented with glucose, have been commonly used in permeability studies. However, these media have been criticized for supporting cell growth, which renders them inappropriate models for the epithelial infection process [29,30]. This raised the need for alternative media that can address this shortcoming and thereby give a better simulation of the intestinal conditions. In order to address this, simulated intestinal fluids (SIF), namely, the Fed State SIF (FeSSIF) originally proposed for evaluation of drug dissolution kinetics [31], and Fasted State SIF (FaSSIF), were evaluated for their compatibility with Caco-2 monolayer. It was found that FeSSIF exhibited cytotoxicity to Caco-2 while FaSSIF was compatible with the Caco-2 monolayer [27,28,32]. Brouwers *et al*., [33] reported that data generated using FaSSIF were similar to that obtained with actual human intestinal aspirates collected in the fasted state. Hence, in order to determine the effect of the recombinant Lbc^InlAB^ on the epithelial infection process of *L*. *monocytogenes in vitro*, we used a Caco-2 cell culture model and FaSSIF as the medium.

## Materials and methods

### Bacterial strains, plasmids and growth conditions

*Listeria monocytogenes* F4244 (serovar 4b, epidemic strain), *Lactobacillus casei* (Lbc^WT^), *L*. *casei* expressing InlAB (Lbc^InlAB^) and LAP (Lbc^LAP^, unpublished), and *L*. *casei* carrying the pLP401T empty vector (Lbc^V^) were used in this study. Cloning and expression of *inlAB* gene of *L*. *monocytogenes* in *L*. *casei* (Lbc^InlAB^) has been reported before [26] where the pLP401T vector [34] contains the origin of replication of *Lactobacillus* and the α-amylase promoter gene (Pamy). The promoter is mannitol inducible, therefore, modified MRS supplemented with 1% mannitol was used to induce the expression of InlAB in *L*. *casei* [21]. The vector also has an anchor peptide (117 aa) gene of *L*. *casei* and transcription terminator of the *cbh* (conjugated bile acid hydrolase) gene (Tcbh). The surface expression of InlAB on Lbc^InlAB^ is envisaged to covalently link to the cell wall/peptidoglycan via anchor peptide and LPxTG motif [26]. *L*. *monocytogenes* culturewas grown in Tryptone Soy broth supplemented with 0.6% yeast extract (TSA-YE) at 37°C for 18 h. Lbc^WT^ was grown in de Man Rogosa Sharpe (MRS) broth while Lbc^V^ and recombinant Lbc^InlAB^ and Lbc^LAP^ strains were grown anaerobically at 37°C for 16 h in MRS broth containing 2 μg/ml erythromycin.

### Preparation of the FaSSIF

The fasted state simulated intestinal fluid (FaSSIF) was prepared as per Dressman *et al*., [35]. Briefly, 0.78 g potassium hydrogen phosphate (KH_2_PO_4_), 3.28 g of potassium chloride (KCl), 5 mM sodium taurocholate (representative bile salt) and 1.5 mM lecithin were suspended in 150 ml distilled water. The pH of the solution was adjusted to pH 6.8 with 1M NaOH or 1M HCl, and then its volume was made up to 200 ml with distilled water. The FaSSIF was sterilized by filtering through a 0.2 μm filter to avoid thermal denaturation of the media components. The FaSSIF was stored at 4°C and then used within 24 h post preparation.

### Adhesion and invasion of Caco-2 cells by recombinant *L*. *casei*

Human colon adenocarcinoma cell line Caco-2 (HTB37; American Type Culture Collection) was cultured in Dulbecco’s modified eagle’s medium (DMEM with high glucose, HyClone^TM^, GE, Logan, UT) supplemented with 10% Fetal Bovine Serum (FBS Atlanta Biologicals, GA) (D10F). The cells were grown in flasks (Greiner- Bio-One) for up to 10–12 days, trypsinized and then seeded in 12 well plates at a density of 1 × 10^5^ cells/well. The plates were incubated at 37°C in the presence of 7% CO_2_ in a cell culture incubator for 10–12 days to allow for monolayer formation and cell differentiation (10^6^ cells/ well) [21,36].

Overnight (18 h) bacteria pre-cultivated in their respective broths were washed twice with PBS, their absorbance adjusted to OD_600_ = 1 and then they were suspended in FaSSIF to a final concentration of 1 × 10^7^ CFU/ml (MOE or MOI, 10). The Caco-2 cell monolayers were washed three times with DMEM and then exposed separately to the *L*. *casei* strains (Lbc^WT^, Lbc^V^, Lbc^InlAB^ or Lbc^LAP^) and *L*. *monocytogenes* and incubated at 37ºC with 5% CO_2_ for 1 h [21]. Excess media were removed and the cell monolayers were washed three times with DMEM. To enumerate the adhered bacterial cells, cell monolayers were treated with 0.1% Triton X-100, incubated at 37ºC for 10 min. For the invasion assay, the monolayers were exposed to *L*. *monocytogenes* and *L*. *casei* and then washed as was done in the adhesion assay, treated with gentamycin (50 μg/ml, 1 h) and with 0.1% Triton X-100 (37ºC, 10 min). The lysed cell suspensions from both adhesion and invasion experiments were serially diluted in PBS before plating on MRS, MRS supplemented with erythromycin (2 μg/ml) and Modified Oxford (MOX) agar for Lbc^WT^, recombinant *L*. *casei*, and *L*. *monocytogenes*, respectively. All the plates were incubated at 37ºC for 24–48 h before bacterial enumeration.

### Determination of *L*. *monocytogenes* exclusion by *L*. *casei*

The competitive exclusion assay was done as per Koo *et al*. [21] with minor modifications. The absorbance of the bacterial cultures was adjusted to OD_600_ = 1 after they were washed twice with PBS, and then they were suspended in FaSSIF to a final concentration of 1 × 10^7^ CFU/ml (MOI, 10). For competitive adhesion, *L*. *monocytogenes* was co-inoculated with each of the *L*. *casei* strains (Lbc^WT^, Lbc^V^, Lbc^InlAB^ or Lbc^LAP^) to Caco-2 cell monolayer and incubated for 1 h. Adherent bacteria were enumerated as before.

In the inhibition of adhesion assay, the Caco-2 cell monolayers were first inoculated with each *L*. *casei* strain and incubated for 1 h. Unbound bacteria were removed by washing of the wells four times using DMEM. *L*. *monocytogenes* was then added to the wells and plates were incubated for one more hour. Adhered bacteria were released and plated as above. For displacement of adhesion, Caco-2 monolayers were first inoculated with *L*. *monocytogenes* and incubated for 1 h. Then unbound bacteria were washed off as in the inhibition of adhesion assay. *L*. *casei* strains were then added to the wells and plates incubated for another 1 h. Adhered bacteria were released and plated on MRS, MRS supplemented with 2 μg/ml of erythromycin and MOX agar plates for enumeration of Lbc^WT^, recombinant *L*. *casei*, and *L*. *monocytogenes*, respectively [21].

### Inhibition of *L*. *monocytogenes* adhesion and invasion by *L*. *casei*

Bacteria were pre-cultivated in their respective broths for 18 h. The bacterial cultures were washed twice with PBS after adjusting their absorbance to OD_600_ = 1, followed by their resuspension in FaSSIF to the final concentration of 1×10^7^ CFU/ml (MOE/MOI, 10). The Caco-2 cell monolayers were washed and then exposed to the *L*. *casei* strains for 1, 4, 16 and 24 h at 37°C in the humidified incubator with 5% CO_2_. Excess medium in the wells containing unbound *L*. *casei* was removed and replaced with 500 μl of *L*. *monocytogenes* suspended in FaSSIF, and the plates incubated for 1 h at 37°C with 5% CO_2_. The cells were then washed thrice using DMEM. To enumerate the adhered bacterial cells, cell monolayers were treated with 0.1% Triton X-100, incubated at 37ºC for 10 min before plating onto the respective microbiological media as already mentioned.

For inhibition of *L*. *monocytogenes* invasion, the Caco-2 cell monolayers were washed three times with DMEM and then exposed to each *L*. *casei* strain for 1, 4, 16 and 24 h at 37°C with 5% CO_2_. Excess *L*. *casei* cells were removed and replaced with 500 μl of *L*. *monocytogenes* suspended in FaSSIF and then incubated for 1 h at 37°C with 5% CO_2_. To remove the non-adhered bacteria, the cell monolayers were washed three times with DMEM and then treated for 1 h with gentamycin (50 μg/ml). The invading bacterial counts were determined by plating as above.

### Caco-2 cytotoxicity assay

To determine Caco-2 cytotoxicity induced by *L*. *monocytogenes* after pre-exposure to *L*. *casei* over time, we performed the LDH assay [21]. The supernatants after infection with *L*. *monocytogenes* for 1 h were collected and used to analyze for lactate dehydrogenase (LDH) enzyme release. Caco-2 cells that were treated with 500 μl of 0.1% Triton X-100 per well were used as positive control while those treated with DMEM were used as the negative control. From the supernatants collected, 100 μl were transferred to the 96-well flat bottom plate in triplicates and was analyzed using Pierce LDH cytotoxicity assay kit (Thermo Scientific, USA) following the protocol from the manufacturer.

### Transcellular translocation of *L*. *casei* strains and subsequent inhibition of *L*. *monocytogenes* transepithelial translocation by recombinant *L*. *casei*

The Caco-2 cells were grown in 12 well trans-well filter inserts (3-μm pore size) for 20–25 days to reach confluence [6]. TEER of Caco-2 cells was quantified using the Millicell ERS system (Millipore, Billerica, MA) and a TEER value of more than 200 was used for all the experiments. Overnight (18 h) bacteria pre-cultivated in their respective broths were used. The bacterial cultures were washed twice with PBS and then suspended in FaSSIF (MOE, 10). For determining baseline translocation by *L*. *casei* or *L*. *monocytogenes*, the cell monolayers were washed three times with DMEM and then the bacteria were added separately to the apical wells, followed by incubation of microwell plates at 37°C with 5% CO_2_ for 2 h. The liquid from the basal well was collected, serially diluted in PBS and then plated for the enumeration of viable cells (CFU/ ml).

For the inhibition of *L*. *monocytogenes* translocation, *L*. *casei* strains were first added to the apical wells and incubated for 1, 4, 16 and 24 h at 37°C with 5% CO_2_. The liquid from the basal wells was collected, serially diluted in PBS and then plated onto MRS plates for enumeration of *L*. *casei* as described. Subsequently, excess *L*. *casei* cells were removed and replaced with 500 μl of *L*. *monocytogenes* suspended in FaSSIF (MOI, 10) and then incubated for 2 h at 37°C with 5% CO_2_. The liquid from the basal wells was removed and serially diluted in PBS and then plated on MOX plates for the enumeration *L*. *monocytogenes*.

### Epithelial tight junction integrity analysis

Transepithelial electrical resistance (TEER) of Caco-2 cells was measured before and after the exposure to the bacteria using the Millicell ERS system (Millipore, Billerica, MA). Furthermore, we analyzed the epithelial tight junction integrity as per Koo *et al*. [21]. After exposure to *L*. *monocytogenes*, the tight junction permeability was analyzed using Dextran^FITC^ (Mr 3–5 kDa; Sigma) permeability through the transwell filter inserts. Fluorescence of the samples collected from the apical and basolateral chambers was read in a SpectraMax Gemini EM fluorescent plate reader (Molecular Devices; Sunnyvale, CA).

### Statistical analysis

All data were analyzed using Prism 7 software (GraphPad Software Inc., United States), and significance was assigned at *p* < 0.05. Where appropriate, Tukey’s multiple comparisons, with p<0.005 as the significant difference was used to identify statistically significant differences.

## Results

### Adhesion, invasion and translocation profiles of *Listeria monocytogenes* (Lm) and *Lactobacillus casei* (Lbc) strains

**Fig 1:**
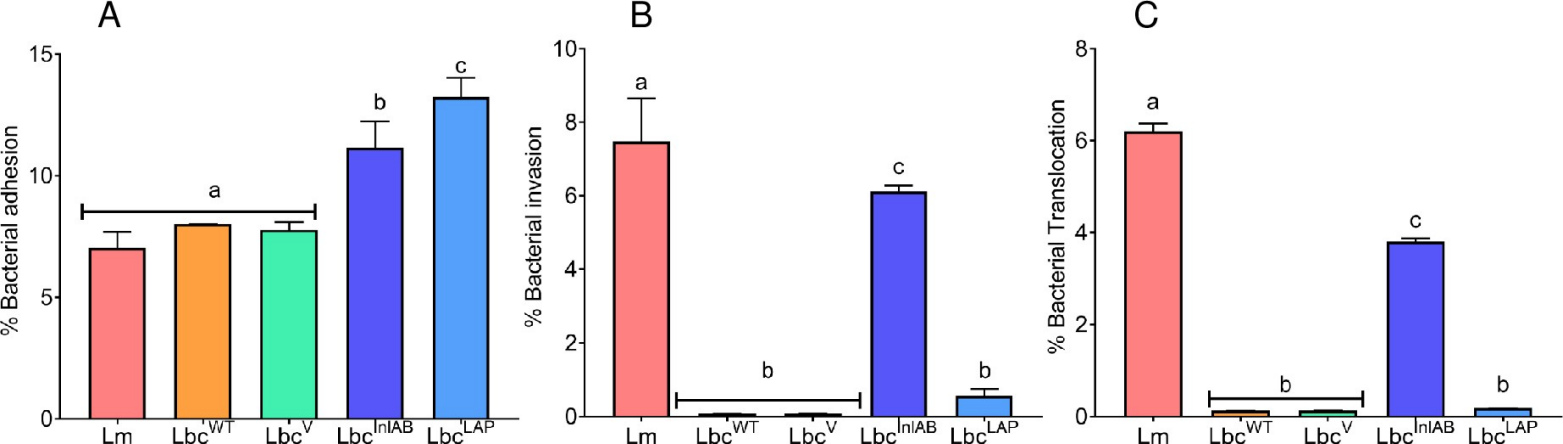
**Adhesion (A), Invasion (B) and Translocation (C) of *Listeria monocytogenes* (Lm) and *Lactobacillus casei* (Lbc**^**WT**^**, Lbc**^**V**^**, Lbc**^**InlAB**^ **and Lbc**^**LAP**^**) to Caco-2 cells.** Percentages were calculated relative to the inocula that were added to the Caco-2 cells. Data are average (SD) of three independent experiments performed in duplicate. Error bars are standard deviations of averages of the three independent experiments. Bars marked with different letters (a, b, c) indicate significant difference at p<0.05.

Probiotics and foodborne pathogens colonize the gastrointestinal tract to exert beneficial effects or cause infection, respectively. It was therefore imperative that we determine how the expression of *L*. *monocytogenes* invasion genes by *L*. *casei* would affect its ability to adhere to, invade and translocate the Caco-2 cells under simulated intestinal conditions when compared to *L*. *monocytogenes*. Fig 1A depicts the adhesion profiles of the *L*. *casei* strains and *L*. *monocytogenes* to Caco-2 cells in simulated intestinal fluid. There were no statistically significant differences in the adhesion of *L*. *monocytogenes* F4244 versus Lbc^WT^ (p = 0.4436) or Lbc^V^ (p = 0.9914) to the Caco-2 cells, which showed adhesion percentages of 7%, 8%, and 7.8%, respectively. Conversely, recombinant *L*. *casei* strains expressing the different genes of *L*. *monocytogenes* adhered to Caco-2 cells at levels significantly higher than those of *L*. *monocytogenes* (p = 0.0002 for Lbc^InlAB^ vs *L*. *monocytogenes* and p <0.0001 for Lbc^LAP^ vs *L*. *monocytogenes*). It is worth noting, adhesion of Lbc^LAP^ was significantly higher than that of Lbc^InlAB^ (p = 0.0229).

Invasion (Fig 1B) and translocation (Fig 1C) profiles of the *L*. *casei* strains and *L*. *monocytogenes* in simulated intestinal conditions were investigated. The strains Lbc^WT^ and Lbc^V^ displayed similar trends in invasion and translocation through the Caco-2 cells, both showing 0.08% and 0.13% for invasion and translocation, respectively. There was an increase in levels of both invasion and translocation by the recombinant *L*. *casei* strains (Lbc^InlAB^ and Lbc^LAP^). Lbc^InlAB^ invaded and translocated through the Caco-2 cells at levels significantly higher compared to those of Lbc^WT^ and Lbc^V^. Invasion and translocation levels for Lbc^LAP^ were not significantly different from those of Lbc^WT^ and Lbc^V^ (p<0.79), but significantly lower than those for Lbc^InlAB^ (p<0.0001). *L*. *monocytogenes* was able to invade and translocate the Caco-2 cell monolayer at significantly higher levels than all the *L*. *casei* strains. What was worth noting is that invasion and translocation of Lbc^InlAB^ through the Caco-2 cells was at significantly higher levels than all the other *L*. *casei* strains.

### Mechanisms of *L*. *monocytogenes* exclusion by the *L*. *casei* strains

**Fig 2:**
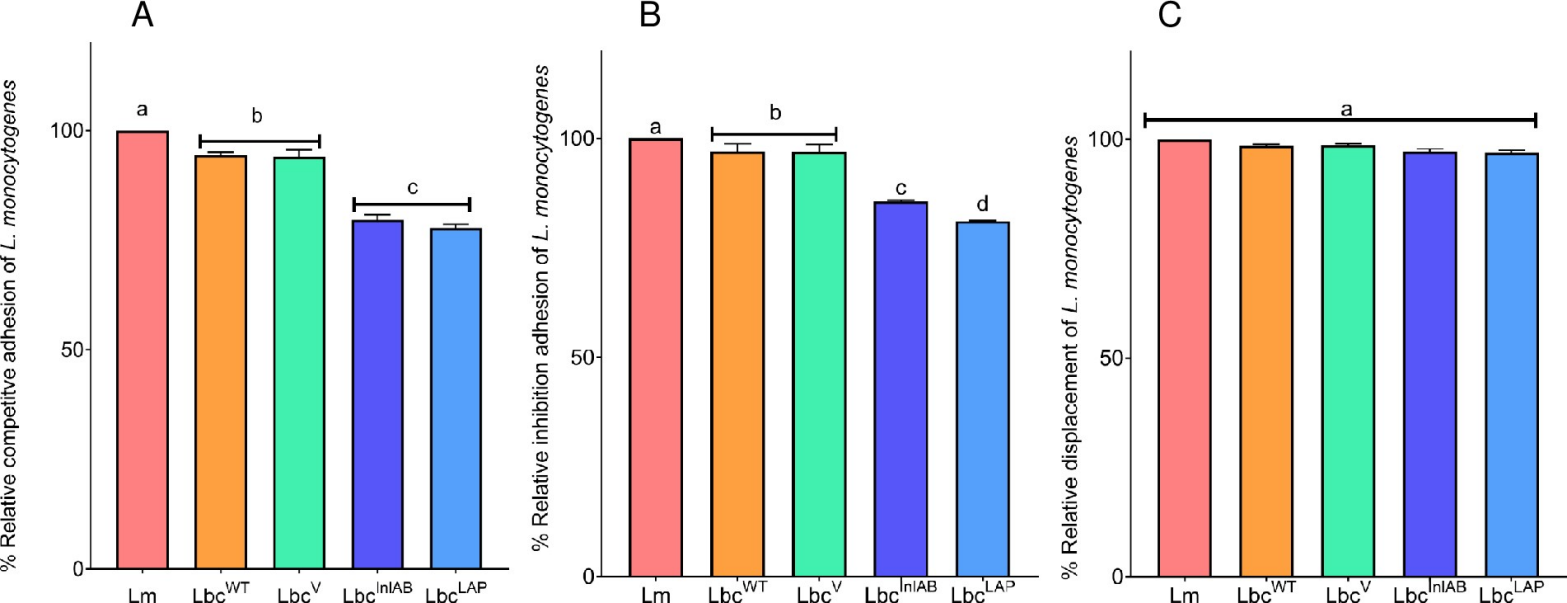
Competitive exclusion of *Listeria monocytogenes* (Lm) adhesion to Caco-2 cells by *L*. *casei* strains (Lbc^WT^, Lbc^V^, Lbc^InlAB^ and Lbc^LAP^), analyzed by three different exclusion mechanisms. (**A**) Competitive adhesion: Caco-2 cells were exposed to *L*. *casei* strains with Lm simultaneously, (**B**) Inhibition of adhesion: Caco-2 cells were pre-exposed to *L*. *casei* strains for 1 h before infection with Lm, and (**C**) Displacement of adhesion: Caco-2 cells were infected with Lm for 1 h before *L*. *casei* strains (1 h). Adhesion of Lm alone to Caco-2 cells was presented as 100% and percentage adhesion was calculated relative to that. Data are averages of three experiments run in duplicates. Error bars are standard deviations of averages of the three independent experiments. Bars marked with different letters (a, b, c, d) indicate significant difference at p<0.05.

Probiotics employ competitive adhesion, inhibition or displacement mechanisms to inhibit or reduce adhesion of pathogens to the intestinal cells. In order to determine which mechanism is employed by *L*. *casei* strains (Lbc^WT^, Lbc^V^, Lbc^InlAB^ and Lbc^LAP^) against *L*. *monocytogenes*, we evaluated each under simulated intestinal conditions (Fig 2). Adhesion of *L*. *monocytogenes* to Caco-2 cells in the absence of the *L*. *casei* strains was recorded as 100% in all the assays and was used to calculate the relative adhesion in the presence of the *L*. *casei* strains.

Fig 2A shows that adhesion of *L*. *monocytogenes* to Caco-2 cells was insignificantly reduced when it was co-inoculated with Lbc^WT^ and Lbc^V^ (p = 0.9941). We recorded reductions of 5.67% and 6% in adhesion of *L*. *monocytogenes* by Lbc^WT^ and Lbc^V^, respectively. When co-inoculated with the recombinant strains (Lbc^InlAB^ and Lbc^LAP^), there was a significant reduction (p< 0.0001) in the adhesion of *L*. *monocytogenes*. There was a 20.48% and 22.34% adhesion reduction by Lbc^InlAB^ and Lbc^LAP^, respectively. Although both Lbc^InlAB^ and Lbc^LAP^ reduced the adhesion of *L*. *monocytogenes*, there was no statistical difference in their reduction levels (p = 0.2620).

In the inhibition of adhesion assay (Fig 2B**)**, adhesion of *L*. *monocytogenes* to the Caco-2 cells was reduced by 2.92% and 3.05% due to their pre-exposure to Lbc^WT^ and Lbc^V^, respectively. The reductions recorded were significant for both Lbc^WT^ (p = 0.0494) and Lbc^V^ (p = 0.0391), however, as expected, there was no significant difference when comparing the inhibition of *L*. *monocytogenes* adhesion by Lbc^WT^ vs. Lbc^V^ (p = 0.4588). Adhesion of *L*. *monocytogenes* was reduced by 18.9% and 14.4% due to pre-exposure of the Caco-2 cells to the recombinant strains Lbc^InlAB^ and Lbc^LAP^, respectively. Interestingly, these recorded reduction levels were significantly higher when compared to the adhesion of *L*. *monocytogenes* alone (p<0.0001). Furthermore, there was a significant difference (p< 0.0033) in the reduction of adhesion between the two recombinant strains, with Lbc^LAP^ showing higher inhibition of adhesion than Lbc^InlAB^. Significant differences (p<0.0001) were also obtained when comparing inhibition of adhesion by Lbc^WT^ or Lbc^V^ versus Lbc^InlAB^ or Lbc^LAP^. In the displacement of adhesion experiment (Fig 2C), there were no significant differences in the adhesion of *L*. *monocytogenes* alone when compared to in the presence of any of the *L*. *casei* strains. Furthermore, there were no statistical differences among all the *L*. *casei* strains in the displacement of *L*. *monocytogenes*. Thus, the results show that inhibition of adhesion is the mechanism of competition used by the recombinant *L*. *casei* strains to reduce interaction of *L*. *monocytogenes* with Caco-2 cells.

### Inhibition of *L*. *monocytogenes* adhesion, invasion, and translocation by *L*. *casei* over time

**Fig 3:**
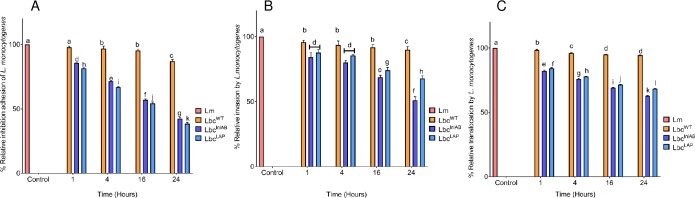
**Inhibition of *Listeria monocytogenes* (Lm) adhesion (A), invasion (B) and translocation (C) by *L*. *casei* strains (Lbc**^**WT**^**, Lbc**^**InlAB**^ **and Lbc**^**LAP**^**) over time.** Caco-2 cells were pre-exposed to the *L*. *casei* strains for 1, 4, 16 and 24 h before infection with Lm for 1 h for adhesion and invasion and 2 h for translocation. Data are averages of three experiments run in duplicates. Error bars are standard deviations of averages of the three independent experiments. For each time point bars marked with different letters (a, b, c, d, e, f, g, h, I, j, k, l) indicate significant difference at p<0.05.

In order to determine how inhibition of *L*. *monocytogenes* adhesion to Caco-2 in FaSSIF will be influenced by duration of pre-exposure of the cell monolayer to *L*. *casei* strains, we investigated adhesion, invasion, and translocation of Caco-2 cells under simulated intestinal conditions by *L*. *monocytogenes* over a 24 h period. The effect of different exposure periods to *L*. *casei* strains on adhesion of *L*. *monocytogenes* to Caco-2 cells is presented in Fig 3A. Adhesion of *L*. *monocytogenes* was more reduced the longer the Caco-2 cells were pretreated with Lbc^WT^, with reductions of 3.29%, 4.51%, and 12.96% recorded for 4, 16 and 24 h pre-exposure times, respectively. Significant reductions due to Lbc^WT^ were recorded after 4 h (p = 0.0007) and 16–24 h (p<0.0001). Improved reductions were obtained due to pre-exposure to recombinant *L*. *casei* strains, with reduced levels of 14.36% and 18.58% after 1 h, as well as of 57.66% and 61.52% after 24 h, recorded for Lbc^InlAB^ and Lbc^LAP^, respectively. Contrary to what was observed for Lbc^WT^, significant reductions in adhesion (p<0.0001) were obtained for Lbc^InlAB^ and Lbc^LAP^ for all exposure periods. Furthermore, even though prolonged exposure to either of the recombinant *L*. *casei* strains enhanced inhibition of adhesion, it was interesting to observe that pre-exposure to Lbc^LAP^ maintained significantly higher reduction levels than Lbc^InlAB^ throughout the 24 h (p<0.0001) period.

Similar trends were observed for invasion (Fig 3B**)** and translocation (Fig 3C) of Caco-2 cells by *L*. *monocytogenes* subsequent to their prolonged pre-exposure to *L*. *casei* strains. Pre-exposure of the Caco-2 cells to Lbc^WT^ for 1 to 16 h showed no significant reduction of invasion (p = 0.3088); however, the 24-h exposure time resulted in a significant reduction (p<0.0001) (Fig 3B). Translocation of *L*. *monocytogenes* was significantly reduced (p<0.0001) by this strain from 1 h up to 24 h pre-exposure times (Fig 3C). No significant reduction in invasion was obtained due to pre-exposure of the Caco-2 cells to Lbc^InlAB^ or Lbc^LAP^ for 1–4 h, while the significant reduction (p<0.0001) was evident for 16 h to 24 h pre-exposure period to these strains. Similarly, when comparing the recombinant *L*. *casei* strains, there were no significant differences at 1 and 4 h pre-exposure, however, they exhibited significant differences after 16 and 24 h pre-exposure (p<0.0001). Pre-exposure to Lbc^InlAB^ and Lbc^LAP^ for 24 h showed significant (p<0.0001) reductions of *L*. *monocytogenes* invasion, with 48.96% and 32.22% reductions recorded for Lbc^InlAB^ and Lbc^LAP^, respectively (Fig 3B).

For translocation assays (Fig 3C), after 24 h of pre-exposure, reductions of 17.81% and 15.67% were recorded for Lbc^InlAB^ and Lbc^LAP^, respectively. Prolonged exposure of the Caco-2 cells to the recombinants showed an even significantly (p<0.0001) enhanced reduction of translocation. Overall, the results indicate that the longer the Caco-2 cells were pre-exposed to *L*. *casei* strains before their infection with *L*. *monocytogenes*, the more adhesion, invasion, and translocation of *L*. *monocytogenes* were reduced.

### Cytotoxicity of *L*. *monocytogenes* to Caco-2 cells in the presence of *L*. *casei* strains

**Fig 4:**
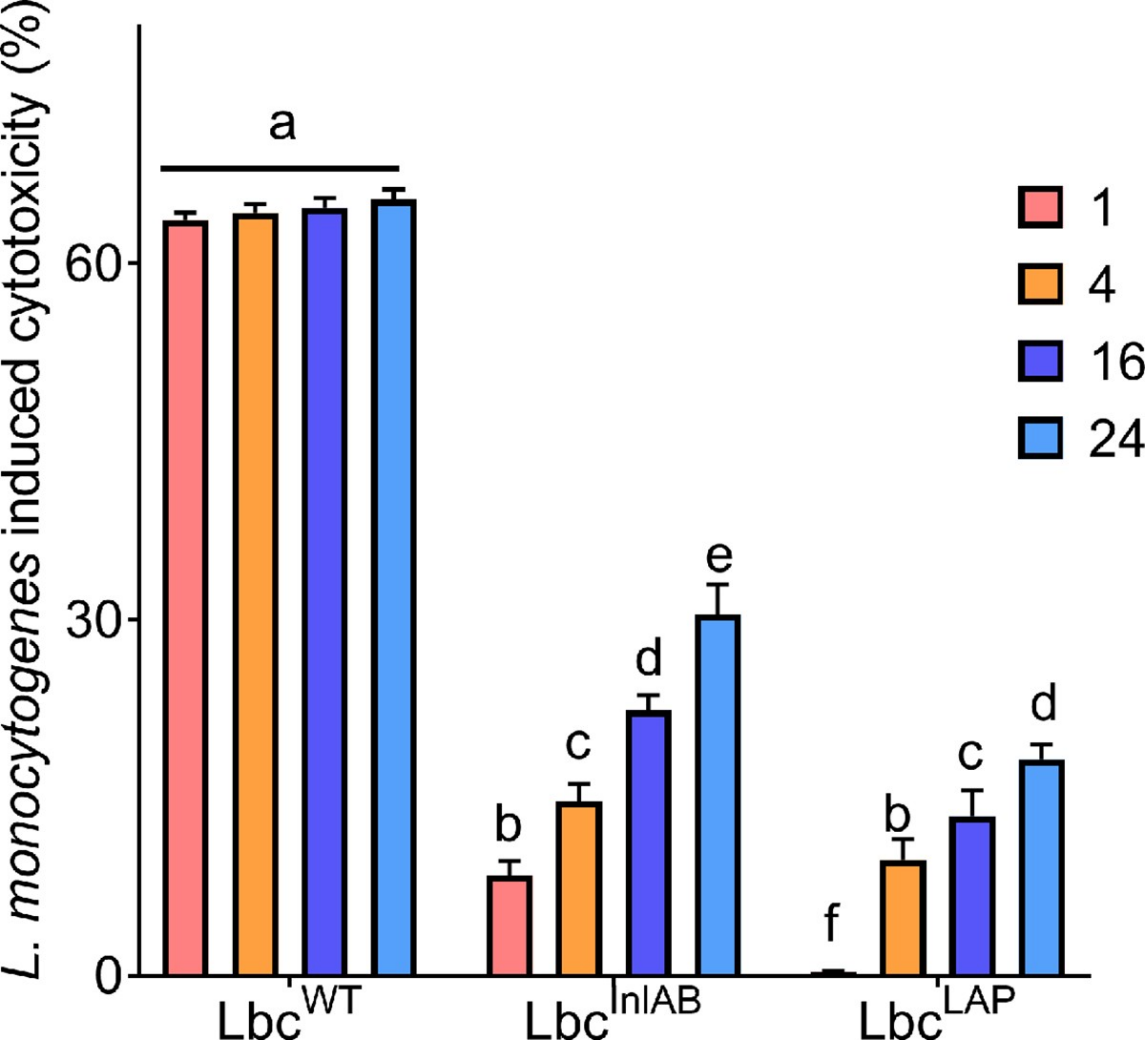
Cytotoxicity of *Listeria monocytogenes* (Lm) on Caco-2 cells pre-exposed to *L*. *casei* over time. Cytotoxicity value for *L*. *monocytogenes* treatment in the absence of *L*. *casei* strains was 70.25%. Data are averages of three experiments run in duplicates. Error bars are standard deviations of averages of the three independent experiments. Bars marked with different letters (a, b, c, d, e, f) indicate significant difference at p<0.05.

*L*. *monocytogenes*-mediated cytotoxicity to the Caco-2 was investigated (Fig 4) using the lactate dehydrogenase (LDH) assay. In the absence of *L*. *casei* strains, *L*. *monocytogenes* treatment for 1 h induced 70.25% cytotoxicity to Caco-2 cells. Pre-exposure of the cells to *L*. *casei* strains resulted in a reduction of cell cytotoxicity. *L*. *monocytogenes* induced 63.7% and 65.42% cytotoxicity levels after 1 h and 24 h, respectively, when Caco-2 cells were pre-exposed to Lbc^WT^ while 8.45% and 30.45% cytotoxicity levels were recorded after pre-exposure to Lbc^InlAB^ for 1 and 24 h, respectively (Fig 4). When the cells were pre-exposed to Lbc^LAP^ for 1 and 24 h, *L*. *monocytogenes* induced only 0.34% and 18.25% cytotoxicity levels for these exposure periods, respectively. These data indicate that recombinant *L*. *casei* strains provide significant protection (p<0.0001) against the cytotoxic effect of *L*. *monocytogenes* than Lbc^WT^.

### Epithelial tight junction integrity analysis

**Fig 5:**
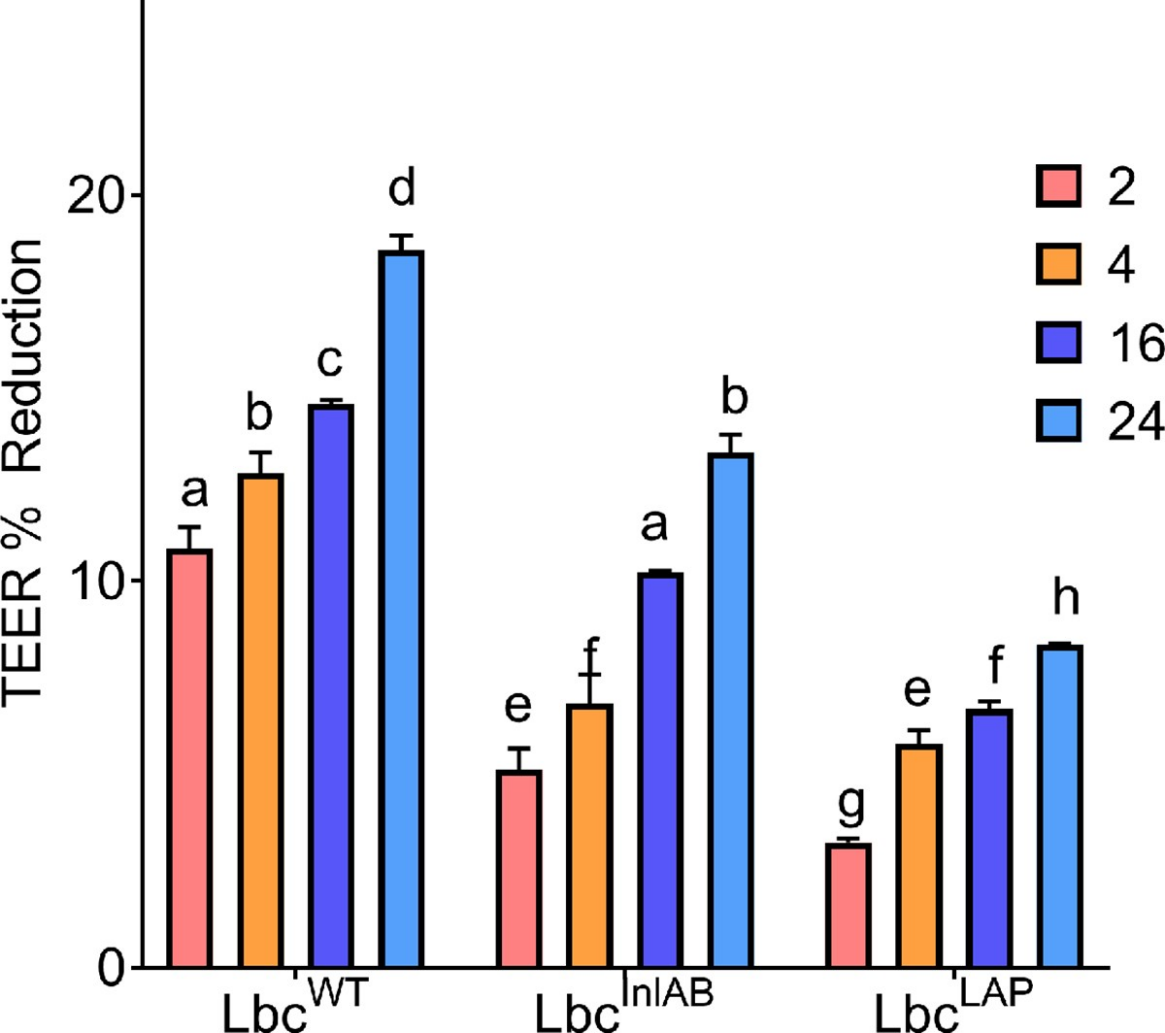
Caco-2 cell permeability analysis using transepithelial electrical resistance (TEER) in simulated intestinal conditions. TEER reduction by *L*. *monocytogenes* in the absence of *L*. *casei* strains was 20.5%. Bars marked with different letters (a, b, c, d, e, f, g, h) indicate significant difference at p<0.05, error bars are standard deviations of average TEER reductions of the three independent experiments.

**Fig 6:**
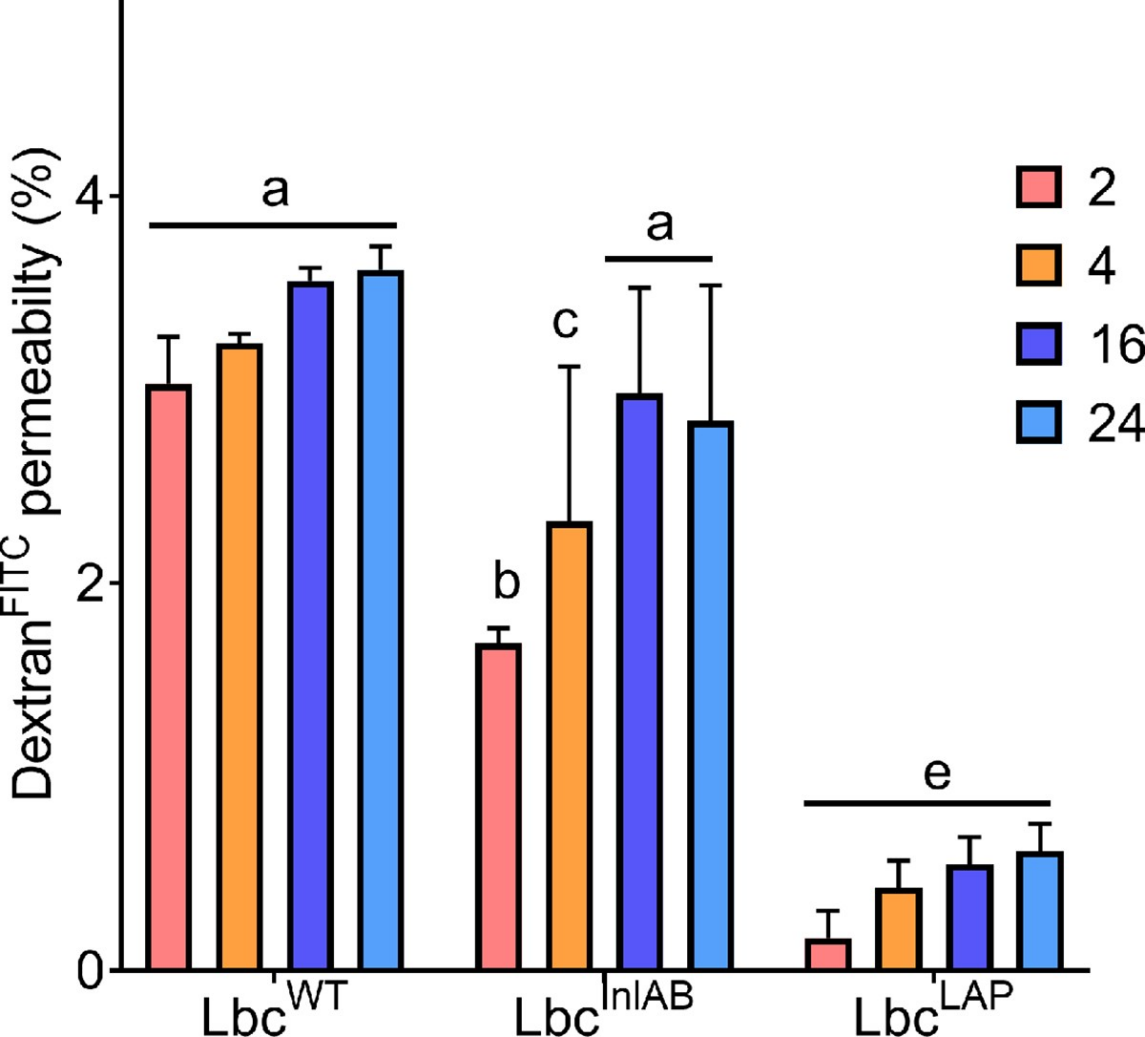
Tight junction integrity analysis using Dextran^FITC^ permeability assays. Dextran^FITC^ recovery after exposure to *L*. *monocytogenes* alone was 3.72± 0.03%. Bars marked with different letters (a, b, c, d, e, f) indicate significant difference at p<0.05, error bars are standard deviations of average Dextran^FITC^ recovered from the three independent experiments.

The integrity of the Caco-2 cells infected with *L*. *monocytogenes* alone or after their exposure to *L*. *casei* strains in the FaSSIF was measured using the transepithelial electrical resistance (TEER) (Fig 5) and Dextran^FITC^ (Fig 6) analyses. The results obtained for both analyses complemented those for cytotoxicity test. When the Caco-2 cells were pre-treated with *L*. *casei* strains for all exposure periods tested, there were lower TEER reduction changes than that of cells treated with *L*. *monocytogenes* alone (Fig 5). TEER reduction changes were also lower due to pre-exposure to recombinant *L*. *casei* strains than that due to their wild type counterpart. When comparing recombinant strains, TEER reductions were lower for Lbc^LAP^ than Lbc^InlAB^. These results showed that under simulated intestinal conditions, recombinant *L*. *casei* strains protected the integrity of tight junctions between the Caco-2 cells, with Lbc^LAP^ showing better protection than Lbc^InlAB^. However, as was also observed for cytotoxicity analysis, prolonged exposure of Caco-2 cells to the probiotics in FaSSIF had negative effects on Caco-2 cells as it resulted in higher TEER reductions when compared to shorter exposure periods. Nevertheless, even after 24 h, the TEER reductions for Caco-2 cells pre-exposed to *L*. *casei* strains were still lower than that of *L*. *monocytogenes* treatment alone.

The results of the Dextran^FITC^ permeability analysis indicated that pre-treatment of the Caco-2 cells with *L*. *casei* strains reduced their permeability induced by *L*. *monocytogenes* since the amount of Dextran^FITC^ recovered from the basal chamber of the transwell plate was always higher for cells infected with *L*. *monocytogenes* alone than that for those pre-exposed to *L*. *casei* strains (Fig 6). Comparing the amount of dye recovered in the basal chamber for cells pre-exposed to *L*. *casei* strains, recombinants (Lbc^InlAB^ and Lbc^LAP^) showed better protection than Lbc^WT^, however, Lbc^LAP^ showed better protection than Lbc^InlAB^. These differences are in agreement with those found in the inhibition of translocation and the TEER reduction tests, meaning that Lbc^LAP^ was better at protecting the integrity of Caco-2 cells under simulated intestinal conditions. The amount of dye recovered increased with an increase in the duration of exposure to *L*. *casei* strains, with levels higher after 24 h than after 2 h for all the strains. These results confirmed observations from the cytotoxicity and TEER reduction assays, which showed that prolonged exposure of Caco-2 cells to the *L*. *casei* strains in FaSSIF affected them negatively.

## Discussion

There is a dire need for safe alternative methods for the prevention or treatment of foodborne diseases due to the ever-increasing development of microbial resistance to antibiotics. Inactivated or attenuated pathogens were initially used as vaccines; however, the risks associated with their use sparked an increased interest in using safe nonpathogenic bacteria as a substitute [15]. These risks include possible reversion of the attenuated pathogen to virulent phenotypes in hosts as well as the possibility for becoming virulent, especially in immunocompromised individuals [15,37]. Probiotics have been reported to offer beneficial health-promoting effects on the host and are generally regarded-as-safe (GRAS), attributes that predispose them as an attractive alternative [38]. The use of probiotics for inhibition of pathogens has been reported in the literature, however, with inconsistent outcomes whereby in certain cases, they have been reported to be less effective against some pathogens. In an effort to eliminate or lessen the limitations of conventional probiotics and enhance the efficacy of probiotics against pathogens, probiotic bioengineering, which is used to design and develop recombinant probiotic strains harboring certain virulence factors of the targeted pathogen, is currently being explored.

We used probiotic *L*. *casei* strain for bioengineering since it does not possess any virulent genes and has been used as probiotic before [39]. We used pLP401T as a vector to clone and express InlAB and this vector has been used before [21,40] and the recombinant *L*. *casei* strains exhibited desired beneficial effects compared to the wild type strain. Furthermore, the recombinant strains did not exhibit any cytotoxic response in the *in vitro* experiments [21,26] including this study suggesting these recombinant strains possibly does not have negative effect. However, animal studies are needed to fully validate their health beneficial effects. For successful application of recombinant probiotic strains, it is necessary to design and construct new suitable expression vectors that will not contain antibiotic markers for selection, preferably integrative vectors.

These genetically engineered probiotics, depending on the virulence genes they express and their mechanism of action can be used as either prophylactics or therapeutics for control of the specific pathogens. Although there have been concerns regarding probiotic bioengineering, it was reported that the probiotics retain their GRAS status even after the expression of heterologous genes [41].

During the infection process, *L*. *monocytogenes* employs the LAP for its adhesion and transcellular migration across the epithelial barrier [5–7] and invasion proteins, InlA and InlB to invade a wider range of mammalian cells [3]. Therefore, in order to construct a probiotic strain with an enhanced ability for targeted control of *L*. *monocytogenes*, we have recently cloned and expressed InlAB into *L*. *casei* which showed enhanced ability to adhere to, invade and translocate through Caco-2 cells *in vitro* compared to that of the wild type strain [26]. Internalins belong to a group of surface exposed leucine rich repeat proteins. They comprise of N-terminal cap domain and also harbour both an N-terminal signal peptide and a C-terminal LPxTG motif followed by a hydrophobic transmembrane region, marking them as extracellular proteins, covalently attached to the bacterial cell wall peptidoglycan [42]. Cloning of *inlAB* using pLP401T allowed anchoring and surface expression of InlAB on the recombinant Lbc^InlAB^ strain [26,34].

One shortcoming of our earlier study is that the experiments were performed *in vitro* using the Caco-2 cells grown and maintained in the standard cell culture media, DMEM supplemented with fetal bovine serum. In its disease progression, *L*. *monocytogenes* has to overcome diverse suboptimal microenvironments that usually constitute the host’s defense system [43] in order to colonize the host GI tract and cause infection. These conditions include but are not limited to low acid in the stomach and high bile concentration in the small intestine. In an effort to better understand the epithelial infection processes of *L*. *monocytogenes* as influenced by the recombinant *L*. *casei* strains, in the current study, we investigated the effect of Lbc^InlAB^ on the interaction between *L*. *monocytogenes* and Caco-2 cells in FaSSIF. Gamboa and Leong [44] reported that SIF has osmolality that is similar to that of human cells thus making this fluid a better medium to be used in *in vitro* intestinal model. The results in Fig 1 shows that expression of InlAB and LAP by the recombinant Lbc^InlAB^ and Lbc^LAP^ exhibited an enhanced adhesion (versus *L*. *monocytogenes* and Lbc^WT^), invasion and translocation as opposed to Lbc^WT^. The increased adhesion of recombinant strains when compared to *L*. *monocytogenes* is potentially due to high plasmid copy numbers in the recombinants. However, it was worth noting that Lbc^LAP^ showed a better adhesion capability as opposed to Lbc^InlAB^, which exhibited superior invasion potentials instead. These results are in correlation with the function of the LAP and InlAB genes in the pathogen adhesion and invasion. Guimarães *et al*. [45] cloned and expressed the invasion gene InlA into *Lactococcus lactis* and reported that the recombinant showed an enhanced ability to invade epithelial cells. In a different study, Koo *et al*. [21] cloned and expressed the LAP into probiotic *Lactobacillus paracasei* and reported that the resultant recombinant strain exhibited enhanced adhesion to the Caco-2 cells. Although these studies reported the enhancement of probiotics through genetic engineering, the cells that they used were maintained in media that supported the growth of the epithelial cells, which is undesirable in such studies. In the current study, we used the FaSSIF, which does not stimulate the growth of epithelial cells, therefore presents a better simulation of the *in vivo* intestinal conditions when compared to the abovementioned studies.

Researchers elsewhere have investigated the intestinal phase of *L*. *monocytogenes* infection process in artificial gastrointestinal fluid broth systems [46–48]. These studies reported on the behavior of *L*. *monocytogenes* when it was introduced on its own to these conditions. In the current study, we investigated the intestinal infection phase of *L*. *monocytogenes* in FaSSIF in the presence of *L*. *casei* strains. We looked at the ability of the *L*. *casei* strains to competitively exclude *L*. *monocytogenes* using three different mechanisms: competitive adhesion, inhibition, and displacement of adhesion, under simulated intestinal conditions (Fig 2**)**. The results revealed that during competitive adhesion and inhibition of adhesion, adhesion of *L*. *monocytogenes* to Caco-2 cells was reduced by the *L*. *casei* strains, with recombinant Lbc^InlAB^ and Lbc^LAP^ exhibiting an enhanced reduction compared to Lbc^WT^. Our results were in agreement with previous studies that reported on the competitive exclusion of pathogens. It has been shown that some probiotics share binding specificities with some pathogens [49,50], making it possible for direct competition between the probiotics with specific pathogens for receptor sites on the host cell [51]. Lee and Puong [52] reported that the inhibition of pathogens by probiotics could be due to the interaction of specific adhesins and receptors present in both probiotic and pathogen, affording the ability to compete for attachment to the same receptors. It has to be highlighted that although our study investigated the intestinal phase of *L*. *monocytogenes* infection processes in presence of recombinant *L*. *casei* strain, it still does not sufficiently simulate the intestinal conditions, specifically with regard to the presence of other intestinal microbiota, which may influence the results currently reported. An efficient platform for deciphering clearer conclusions pertaining to the actual effects of the recombinant *L*. *casei* strain on *L*. *monocytogenes* intestinal infection phase will be through the animal studies in these missing inhabitants of the GIT will be present. Converse to the results for competitive adhesion and inhibition of adhesion, all the *L*. *casei* strains failed to displace *L*. *monocytogenes* cells that had already adhered to Caco-2 cells similar to previous reports [53,54]. This observation negates the suitability of the recombinant *L*. *casei* strains as a therapeutic intervention for *L*. *monocytogenes* infection.

Previously, Barmpalia-Davis *et al*. [55] reported that artificial gastrointestinal conditions closely simulate the dynamics of GIT. Bernbom *et al*. [56] reported that in order to eliminate the influence of the indigenous microflora during pathogenesis studies and thereby simplify results interpretation, *in vitro* models of the intestinal system can be used. Taking these studies into consideration, we further examined how the presence of *L*. *casei* strains affected the different stages in the infection cycle of *L*. *monocytogenes* under simulated intestinal conditions over various exposure times (Fig 3**)**. Prolonged exposure of the Caco-2 cells to *L*. *casei* strains showed enhanced inhibition of *L*. *monocytogenes*. Koo *et al*. [21] also reported that prolonged exposure of the Caco-2 cells to recombinant *L*. *paracasei* expressing LAP showed enhanced inhibition of *L*. *monocytogenes* adhesion, invasion, and translocation. Furthermore, it was worth noting that in all stages of infection the recombinant *L*. *casei* strains were better at inhibiting *L*. *monocytogenes* than Lbc^WT^. Consequent to studying the inhibition of translocation, we also monitored the tight junction integrity using electrical resistance (Fig 5), and Dextran^FITC^ (Fig 6**)** permeability assays in the presence of *L*. *casei* strains. In agreement to the results observed for the inhibition of adhesion, invasion, and translocation, tight junction integrity of Caco-2 cells was better preserved in the presence of *L*. *casei* strains under simulated intestinal conditions. *L*. *monocytogenes* translocation has been reported to potentially occur in the stomach [57], the small intestine [6,58–60] and the large intestine [61] in murine models. The enhanced protection of the tight junction by the recombinant *L*. *casei* strains in simulated intestinal conditions will result in a reduction of *L*. *monocytogenes* translocation, therefore, inhibiting *Listeria* infection. Our findings reported here are likely to be a closer reflection of the effects of the recombinant Lbc^InlAB^ on intestinal disease progression of *L*. *monocytogenes in vivo*. Nevertheless, this does not eliminate the need for animal studies.

In summary, the current study shows that the recombinant *L*. *casei* strain expressing InlAB can minimize the interaction of *L*. *monocytogenes* with the Caco-2 cells under simulated intestinal conditions. Additionally, it shows that different stages (adhesion, invasion, and translocation) of the *L*. *monocytogenes* infection cycle can be targeted depending on the virulence genes cloned and expressed. Future *in vivo* studies are recommended to confirm the purported effects of the *L*. *casei* strains under the actual conditions. These studies should address among others, safety issues associated with the use of the recombinant strains and investigate the apparent reduction of disease progression and/or disease severity in the presence of other GIT microorganisms.

## Supporting information

S1 Table Raw data for adhesion to Caco-2 cells. (XLSX)

S2 Table Raw data for invasion of Caco-2 cells. (XLSX)

S3 Table Raw data for translocation through Caco-2 cells. (XLSX)

S4 Table Raw data for competitive adhesion of *L. monocytogenes*. (XLSX)

S5 Table Raw data for inhibition of adhesion *L. monocytogenes*. (XLSX)

S6 Table Raw data for displacement of adhesion *L. monocytogenes*. (XLSX)

S7 Table Raw data for inhibition of adhesion over time. (XLSX)

S8 Table Raw data for inhibition of invasion over time. (XLSX)

S9 Table Raw data for inhibition of translocation over time. (XLSX)

S10 Table Raw data for summary cytotoxicity, TEER and FITC. (XLSX)
